# Mortality Prediction in Patients With Breast Cancer by Artificial Neural Network Model and Elastic Net Regression

**DOI:** 10.34172/jrhs.2025.173

**Published:** 2024-12-25

**Authors:** Anis Esmaeili, Ali Karamoozian, Abbas Bahrampour

**Affiliations:** ^1^Department of Biostatistics and Epidemiology, School of Public Health, Kerman University of Medical Sciences, Kerman, Iran; ^2^Modeling in Health Research, Institute for Future Studies in Health, Kerman University of Medical Sciences, Kerman, Iran

**Keywords:** Elastic net regression, Artificial neural network, Breast cancer

## Abstract

**Background:** Breast cancer (BC) is the most common cancer in women, and it is important to identify models that can accurately predict mortality in patients with this cancer. The aim of the present study was to use the elastic net regression and artificial neural network (ANN) models in diagnosing and predicting factors affecting BC mortality.

**Study Design:** A cross-sectional study.

**Methods:** The data of 2,836 people with BC during 2014-2018 were analyzed in this study. Information was registered in the cancer registration system of Kerman University of Medical Sciences. Death status was considered the dependent variable, while age, morphology, tumor differentiation, residence status, and residence place were regarded as independent variables. Sensitivity, specificity, accuracy, area under the *receiver operating characteristic* curve (AUC), precision, and F1-score were used to compare the models.

**Results:** Based on the test set, the elastic net regression determined factors affecting BC mortality (with sensitivity of 0.631, specificity of 0.814, AUC of 0.629, accuracy of 0.792, precision of 0.318, and F1-score of 0.42) and ANN did so (with sensitivity of 0.66, specificity of 0.748, AUC of 0.704, accuracy of 0.738, precision of 0.265, and F1-score of 0.37).

**Conclusion:** The sensitivity and AUC of the ANN model were higher than those of the elastic net regression, but the specificity, accuracy, precision, and F1-score of the elastic net were higher than those of the ANN. According to the purpose of the study, two models can be used simultaneously. Based on the results of models, morphology, tumor differentiation, and age had a greater effect on death.

## Background

 Cancer is the main cause of death in developed countries and the second main cause of death in developing countries. In Iran, cancer is the second largest group of chronic non-communicable diseases and the third most common cause of death after heart disease, accidents, and other natural phenomena. Breast cancer (BC) is the most prevalent malignancy among the female population worldwide.^1,2^ Women’s BC is the leading cause of cancer incidence, with about 2.3 million new cases, accounting for 11.7% of all cancer cases (1 in 4 cancer cases). In Iran, BC is the most frequent cancer among females. According to the World Health Organization prediction, up to 2.3 million women will be diagnosed with BC by 2050.^3^ In recent years, data mining methods have been used to diagnose diseases. Data mining is generally the process of finding significant structures in data. Data mining finds its origins in the realms of statistics, mathematics, machine learning, artificial intelligence, and business. Machine learning is a sub-form of artificial intelligence that provides new insight or knowledge via extracting functional patterns and applicable models from large volumes of the raw dataset. These techniques can discover patterns and relationships between them from complex datasets, while they can effectively predict future outcomes of cancer type.^4,5^

 The most considered subject in medical research is human health, and available methods of modeling and prediction in classic statistics are not practical enough due to their limitations; thus, applying these sorts of models leads to some problems and limitations. For example, in logistic regression, the assumption of independence of errors and variables is essential. Under this condition, if the relevant data are complicated, the model’s assumptions may not be true anymore. Over the last decades, data analysts have used various survival methods, such as the Cox regres­sion hazard or the parametric regression models, for analyzing survival data sets. However, the human body is a complex biological system, and most clinical characteristics exhibit a multidimensional and non-linear rela­tionship. Thus, it is difficult to predict the mortality of cancer with a conventional statistical technique. One way to solve such problems is using an artificial neural network (ANN); it is a novel computer model inspired by the function of the human brain that can build non-linear statistical models to assess complex biological systems.^6,7^ In this study, an ANN model has been developed for diagnosing and predicting factors affecting BC mortality.

 Regression analysis is one of the most widely used methods for fitting models to data. The least squares method is one of the simplest and most efficient estimation methods. When some variables are a linear combination of one or more other variables or the number of predictors is large, the least squares method does not show reliable performance. Among the damages to the model when using these methods are the lack of stability, low prediction accuracy, incorrect selection of variables, and poor prediction and interpretation. Penalization techniques have been proposed to improve least squares, especially when the correlations among variables are high.^8,9^ Ridge, Lasso, and elastic net regression are kinds of penalization methods. Ridge regression always keeps all the predictors in the model. Lasso regression does both continuous shrinkage and automatic variable selection simultaneously, but if there is a group of variables among which the pairwise correlations are extremely high, the Lasso tends to select only one variable from the group and does not care which one is selected, leading to choosing one and ignoring others. Elastic net regression simultaneously does automatic variable selection and continuous shrinkage. Moreover, it can select groups of correlated variables.^9^ Elastic net regression can achieve a better trade-off between bias and variance than lasso and ridge regression by tuning the regularization parameters. Furthermore, this type of regression can be applied to various types of data, such as linear, logistic, or Cox regression models.

 Numerous studies have compared neural network in predicting mortality and other consequences of various diseases with other prediction models. NN and elastic net regression have also been used in various studies to diagnose cancer. However, no study has so far been conducted to predict BC death using NN and elastic net regression. Thus, this study aimed to compare the performance of NN and elastic net regression in predicting the mortality of BC and determining factors affecting it.

## Methods

 The data of this study were obtained from 2836 patients (whose information was registered in the cancer registration system of Kerman University of Medical Sciences) with BC during 2014-2018. Death status was considered a dependent variable, and its value was determined according to the presence or absence of a death certificate in patients’ files when extracting information from the system. Around 88.1% (2498 people) survived of all patients, while the remaining 11.8% (338 people) died. Tumor differentiation, place of residence, and residence status, respectively, had 26%, 1.5%, and 0.9% missing values that were estimated by the expectation–maximization algorithm. Considering that the mortality rate was unbalanced and about 12% of the subjects would die, 12% was considered the probability cut-off point for calculating the models. Age, morphology, tumor differentiation, place of residence, and residence status were considered independent variables.

 The hold-out cross-validation method was used in this study. Randomly 70% of the data were regarded as the training set, and the remaining 30% were considered the test set. Then, the model was trained on the training set and finally validated on the test set. The hold-out method is usually used on large datasets as it requires training the model only once. Additionally, to prevent overfitting, the dataset must be randomly separated as the training set and test set. The proportion of deaths is consistent across both sets.

 Elastic net regression and ANN models (using R software) were fitted to the data to predict BC mortality. Sensitivity, specificity, area under the receiver operating characteristic curve, and accuracy were used to compare the predictive power of the models; these are key performance metrics utilized to evaluate classification models in machine learning. If the datasets are imbalanced (similar to the present study), precision, recall, and F1-score are good criteria to compare models. They were reported for a more comprehensive evaluation.

###  Artificial neural network

 ANNs have been developed as the generalizations of mathematical models of biological nervous systems. The basic processing elements of NNs are called artificial neurons or nodes. In a simplified mathematical model of the neuron, the effects of the synapses are represented by connection weights that modulate the effect of the associated input signals, and the nonlinear characteristic exhibited by neurons is represented by a transfer function. The neuron impulse is then computed as the weighted sum of the input signals, transformed by the transfer function. The learning capability of an artificial neuron is achieved by adjusting the weights in accordance with the chosen learning algorithm.

 The basic architecture consists of input, hidden, and output layers.^10^ The size of the input layer is equal to the number of input features, and that of the output layer is equivalent to the number of output classes. In addition, the adjustable parts in the NN are the number and composition of the hidden layer(s); researchers generally use the trial and error method for this purpose.^11^

 The multilayer perceptron NN with the error back-propagation algorithm is utilized for prediction. There are two calculation paths in this algorithm. The first one is the forward path, in which the activation functions work on each neuron. The other is the backward path, in which the error vectors are returned from the last layer to the first. The mean square error index can be employed to stop repeating the algorithm.^12^ The activation function is selected based on the specific need for solving the problem. Some functions are used more, including identity, Sigmoid, Softmax, and hyperbolic tangent functions.^13^

 These methods need two distinct sets. The first part is the training set that helps learn patterns presented in the data, and the second part is the testing set used to validate the network. Therefore, in the first part, the data were randomly divided into two training and testing sets. In this study, different networks were trained by changing the number of nodes in the hidden layer, and then the performance of these networks was compared by the mean square error in the testing set. Overall, 120 ANN models were fitted with 1–10 hidden nodes. Finally, the optimal model was selected according to the lowest error. The networks were trained by the back-propagation algorithm with a learning rate in three ranges (0.01, 0.05, and 0.1) and a momentum value in the range of 0.8–0.95 on the training set. A three-layer perceptron network (6 input, 2 output, and 5 hidden nodes) was used, with a learning rate of 0.01, momentum of 0.8, error-back propagation algorithm, and gradient reduction optimization. The activation function in the hidden layer was the hyperbolic tangent, and the layer was Softmax in the output.

 ANN2 package in R (4-2-1) software was employed to fit this model.

###  Elastic net regression

 In linear regression, the least squares method is utilized to estimate model parameters. The ordinary least squares (OLS) estimates are obtained by minimizing the residual sum of squares. When some variables are a linear combination of one or more other variables or the number of predictors is large, OLS often does poorly in prediction and interpretation.

 Penalization techniques have been proposed to improve OLS. The elastic net is a novel shrinkage and selection method that produces a sparse model with good prediction accuracy while encouraging a grouping effect. The elastic net simultaneously does automatic variable selection and continuous shrinkage and can also select groups of correlated variables. Its estimation is obtained by minimizing the following equation:


$$\begin{array}{l} L\left(\lambda_{1},\lambda_{2},\beta \right)=\;∥(y-X\beta {∥}^{2}+\lambda_{2}∥\beta {∥}_{2}^{2}+\lambda_{1}∥\beta {∥}_{1}\\ \hat{\beta }=argmin\left\{L\left(\lambda_{1},\lambda_{2},\beta \right)\right\} \end{array}$$

Where


$$\begin{array}{l} {\left\|\beta \right\|}_{1}=\sum_{j=1}^{p}\left|\beta_{j}\right|\\ {\left\|\beta \right\|}_{2}^{2}=\sum_{j=1}^{p}\beta_{j}^{2} \end{array}$$

The elastic net estimator is a two-stage procedure. It is another regularization and variable section method that includes a tuning parameter α ≥ 0 and is the combination of two methods (Lasso and Ridge). For each fixed λ_2_, first, the ridge regression coefficients are found, and then the Lasso-type shrinkage is obtained along the Lasso coefficient solution paths. It appears to incur a double amount of shrinkage. Double shrinkage does not help reduce the variances much and introduces unnecessary extra bias compared with pure Lasso or ridge shrinkage.^9^ The elastic net can be defined as:


$${\hat{\beta }}^{elastic}=\left(1+\frac{\lambda_{2}}{n}\right)argmin∥(y-X\beta {∥}^{2}+\lambda_{2}∥\beta {∥}_{2}^{2}+\lambda_{1}{\left\|\beta \right\|}_{1}$$

On setting $\alpha =\frac{\lambda_{2}}{\lambda_{2}+\lambda_{1}}$ the estimator of the equation will be similar to the minimizer of:


$$\begin{array}{l} {\hat{\beta }}^{elastic}=\left(1+\frac{\lambda_{2}}{n}\right)argmin∥(y-X\beta {∥}^{2}+\lambda_{2}∥\beta {∥}_{2}^{2}+\lambda_{1}{\left\|\beta \right\|}_{1}\\ s.t\left(1-\alpha \right)∥\beta {∥}_{1}+\alpha ∥\beta {∥}_{2}\;\leq t \end{array}$$

where *t* is a user-specified parameter, and $\left(1-\alpha \right)\;\;∥\beta {∥}_{1}+\alpha ∥\beta {∥}_{2}^{2}$ is the elastic net and is the convex combination of Lasso and Ridge penalties. For the elastic net, α ∈(0,1), and the elastic net simplifies to simple ridge regression when *α*= 1 and to the Lasso when *α*= 0 ^14^.

 MASS, e1071, and caret packages in R software were used to fit this model.

## Results

 Independent variables are presented in Table 1. According to the clinician, the risk of BC is 50 years. People were divided into two age groups (over 50 years and under 50 years). Table 2 provides the number of observations and predictions by two models.

**Table 1:** Independent variables used in two models to predict the mortality of breast cancer patients

**Independent variables**	**Dead**		**Alive**		**Total**	
	**Number**	**Percent**	**Number**	**Percent**	**Number**	**Percent**
Age (year)						
< 50	133	39.3	1304	52.2	1437	50.7
≥ 50	205	60.7	1194	47.8	1399	49.3
Tumor differentiation						
Good	276	81.7	1941	77.7	2217	78.2
Week and bad	62	18.3	557	22.3	619	21.8
Morphology						
Neoplasm	210	62.1	461	18.5	671	23.7
Infiltrating	117	34.6	1770	70.9	1887	66.5
Other	11	3.3	267	10.7	278	9.8
Residence status						
Urban	295	87.3	2200	88.1	2495	88
Rural	43	12.7	298	11.9	341	12
Residence place						
Kerman	187	55.3	1239	49.6	1426	50.3
Other cities	151	44.7	1259	50.4	1410	49.7

**Table 2:** Observations and prediction by artificial neural network and elastic net regression (confusion matrix)

**Model**	**Alive**	**Dead**
Elastic net		
Not dead	609	139
Dead	38	65
Neural network		
Not dead	560	188
Dead	35	68

 The elastic net regression model was fitted to the training data set using R software. The cut-off point was determined based on reality, considering that 12% of the subjects would die. In addition, 12% was considered the probability cut-off point for calculating the elastic net regression model. Model coefficients for each variable are summarized in Table 3. According to the data, the variables of tumor differentiation, residence status, morphology, and age had a positive effect on BC mortality. In other words, mortality was higher in urban people with neoplasm morphology, and weak and bad degrees of differentiation who were above 50 years. The variable residence place had a negative effect on BC mortality; in other words, BC mortality was lower in other cities than in Kerman. Based on the classification Table 2 for the elastic net regression model, for the test dataset, the sensitivity, specificity, area under the ROC curve (AUC), and accuracy were 0.631, 0.814, 0.629, and 0.792, respectively.

**Table 3:** Parameter estimates of elastic net regression model

**Variables**	**Level**	**Coefficient**
Age	Over 50	0.15
Tumor differentiation	Week and bad	0.21
Morphology	Neoplasm	1.33
Morphology	Infiltrating	0.33
Residence status	Urban	0.07
Residence place	Other cities	-0.13

**Figure 1:**
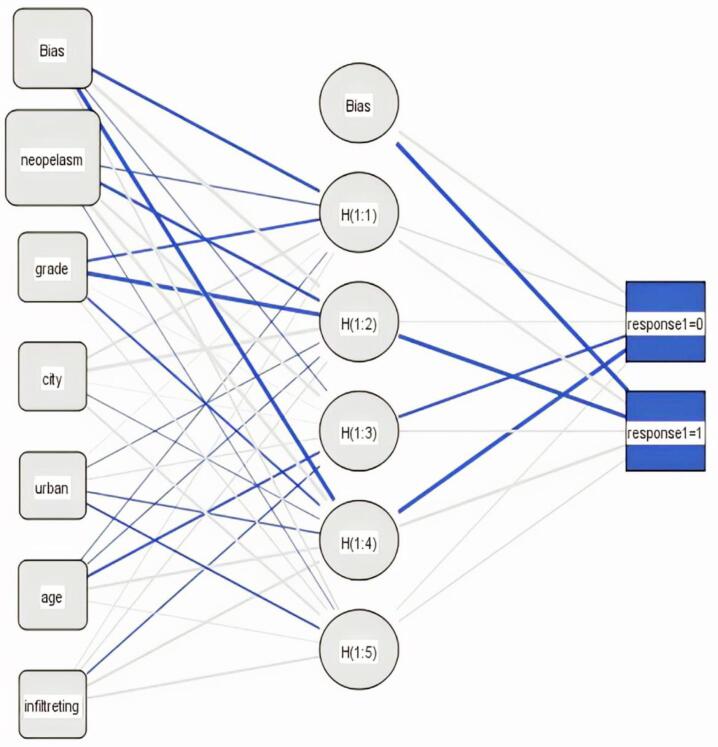
Schematic diagram of the artificial neural network model

Table 4 presents the importance of independent variables in the ANN model. In the three-layer ANN, morphology, degree of tumor differentiation, age, residence place, and residence status had the most effects on BC mortality, respectively. Based on the classification of Table 2 for theANN model, for the test dataset, the sensitivity and specificity were 0.660 and 0.748, respectively. In addition, AUC and accuracy were 0.704 and 0.738, respectively. The NN model was used to obtain the 3-layer network and the relative weights of neurons for the prediction of BC mortality. The multi-layer perceptron network included 6 input neurons, 1 bias neuron in the input layer, 5 hidden neurons, 1 bias neuron in the hidden layer, and 2 output neurons (Figure 1).

**Table 4:** The importance of independent variables on breast cancer mortality in the artificial neural network model

**Variables**	**Level**	**Importance**	**Normalized Importance (%)**
Age (year)	Over 50	0.101	17.6
Tumor differentiation	Week and bad	0.103	18.0
Morphology	Neoplasm	0.572	100
Morphology	Infiltrating	0.077	13.4
Residence status	Urban	0.062	10.8
Residence place	Other cities	0.085	14.8

 For cases where the datasets were imbalanced, precision, recall, and the F1-score were reasonable values (Table 5).

**Table 5:** Evalution criteria of two models

**Criteria**	**Elastic net**	**Neural network**
Sensitivity	0.631	0.660
Specificity	0.814	0.748
Accuracy	0.792	0.738
Area under the ROC curve	0.629	0.704
Precision	0.318	0.265
Recall	0.631	0.660
F1-score	0.423	0.378

*Note*. ROC: Receiver operating characteristic

## Discussion

 The present study compared the elastic net regression and ANN models in predicting BC mortality in Kerman Province in the south of Iran. For the elastic net regression andANN model, sensitivity or recall and specificity were 0.631 and 0.660, as well as 0.814 and 0.748, respectively. Further, AUC and accuracy for the elastic net regression and the ANN model were 0.629 and 0.704, as well as 0.792 and 0.738, respectively. Moreover, precision was 0.318 and 0.265 for the elastic net regression and the ANN model, and the F1-score was 0.423 and 0.378 for both models, respectively. Therefore, the sensitivity and AUC indices of the ANN model were higher in predicting death than the elastic net regression, but the specificity, accuracy, precision, and F1-score indices of the elastic net regression were higher than the ANN model. However, overfitting is a major problem in ANN applications. In large datasets, to prevent overfitting, the dataset must be separated randomly as the training set and test set. After the learning process of ANN by using the training set, the model should be tested in unseen data, a test set, and sometimes a validation set. For the small datasets, the k-fold cross-validation model is suggested to avoid overfitting.

 According to the importance of cancer disease, access to models that can accurately predict patient mortality is highly important. In this regard, several studies have been conducted in different countries. Biglarian et al used the ANN and parametric models (exponential, Weibull, normal, lognormal, logistic, and log-logistic models) to predict the survival status of gastric cancer patients. The AUC for the ANN model was higher than that of the Weibull model,^15^ which is consistent with the result of the present study. Ahmadi and Hoshang Talebi investigated the performance of Lasso, Lars, elastic net, scar, and nonnegative garrote regression methods. this study considered elastic net regression to be suitable in every way compared to other models, and this method had more predictive accuracy than other methods.^16^ It is in line with the results of the present study. The results of the study by Huang et al demonstrated that the ANN model for prediction mortality in BC patients had a significantly higher performance than Cox regression, and multiple logistic regression also had higher overall performance indices.^17^ In the current study, however, the specificity and accuracy of the ANN were less than those of another model. Garcia-Carretero et al compared logistic regression, Lasso, and elastic net methods; the results revealed that penalized regression had a better performance for prediction.^18^ In a study, Liang et al used nine prediction models, including elastic net regression and ANNs. Eventually, logistic regression, elastic net, and ridge models showed the highest overall predictive power compared to other models, and the AUC of these models was more than that of the other models,^19^ which contradicts the results of the present study.

 The contradiction between the results of the studies can be due to the difference in the selection of influential variables in prediction survival status, the different structure of the data, the type of relationship between the independent variables and the response variable, or the different nature of the diseases. One of the limitations of this study was the low number of variables registered in the cancer registration system of Kerman University of Medical Sciences, and it was impossible to access more variables. It is recommended that a similar study be conducted that uses more variables such as a family history of BC, eating habits (healthy or unhealthy), marital status, income status, and the like in the models. In future studies, it is suggested that other machine learning methods such as random forest, Naive Bayes, support vector machines, and the like be applied and compared for different datasets and large amounts of data.

**Highlights**

- The sensitivity and area under the receiver operating characteristic curve of the artificial neural network (ANN) model were higher than those of the elastic net regression.
- The specificity and accuracy of the elastic net regression were higher than those of the ANN.
- Based on the results of both models, morphology, degrees of tumor differentiation, and age had a greater effect on death.
- Mortality increased in urban people with neoplasm morphology, as well as weak and bad degrees of differentiation, who were over 50 years old and lived in Kerman.

## Conclusion

 According to the ANN model, morphology, tumor grade, and age had noticeable effects on cancer mortality. The coefficients of the elastic net regression model showed that mortality increased in urban people with neoplasm morphology, as well as weak and bad degree of differentiation, who were > 50 years old and lived in Kerman. Consequently, it can be controlled effectively by early diagnosis at a younger age, followed by effective treatment.

 The sensitivity (recall) and AUC of the ANN model were calculated more than those of the elastic net regression, but the specificity, accuracy, precision, and F1-score of the elastic net regression were determined to be more than those of the ANN model. According to the purpose of the study, it is recommended that one of the two models be used in future studies. Considering that precision focuses on minimizing false positives, it is recommended that the elastic net model is utilized in future research.Recall should focus on minimizing the chance of missing positive cases, and it is highly important in domains such as medical (e.g., identifying cancer); in this case, ANN is more appropriate.

## Acknowledgements

 The authors thank the cancer registration system of Kerman University of Medical Sciences for providing the information.

## Competing Interests

 The authors confirm that they have no conflict of interests to declare.

## Ethical Approval

 None of the data are published in the name of the participants.

## Funding

 This research received no external funding.
